# The dose-response relationship between physical activity and school scoliosis screening positive in children and adolescents: a preliminary cross-sectional study

**DOI:** 10.3389/fped.2026.1742234

**Published:** 2026-05-14

**Authors:** Chen Chen, Yang Yang, Zhen Chen, Lingyan Yuan

**Affiliations:** 1 School of Physical Education, Sports Kinesiology, Shanghai Normal University, Shanghai, China; 2 Department of Rehabilitation Medicine, Renji Hospital Affiliated to Shanghai Jiao Tong University School of Medicine, Shanghai, China

**Keywords:** adolescents, children, dose-response relationship, physical activity, scoliosis screening positive

## Abstract

**Background:**

While physical inactivity is an established risk factor for adolescent idiopathic scoliosis (AIS), dose-response relationships for physical activity (PA) parameters remain unquantified. This study examined associations between PA dose (amount, duration, frequency, type) and scoliosis screening positive (SSP), exploring optimal thresholds.

**Methods:**

In this cross-sectional analysis, 1,051 students (mean age 10.69 ± 2.45 years) from 8 Shanghai schools underwent scoliometer screening (ATR ≥5° defined as SSP). PA was assessed via validated The Physical Activity Questionnaire for Older Children(PAQ-C) questionnaire[scores≤2: light PA(LPA), scores2-3: moderate PA(MPA), scores > 3: vigorous PA(VPA)]. Logistic regression modeled scoliosis risk across PA strata. Restricted cubic splines (RCS) characterized dose-response curves. Subgroup analyses explored age-specific effects.

**Results:**

In two multivariable-adjusted models, both LPA and VPA demonstrated significantly elevated risks for SSP compared to MPA (adjusted OR = 3.30, 95% CI 1.92–5.68 and OR = 3.65, 95% CI 2.14–6.24, respectively), revealing a U-shaped dose-response relationship with risk nadir at PAQ-C scores≈2.7(p < 0.05). Exercise frequency <3 sessions/week (OR = 4.68, 95% CI 2.75–7.98), session duration <∼54 min or >90 min (OR = 3.43 and 2.75), and cumulative participation >2 years (OR = 3.37, 95% CI 1.89–6.01) were positively associated with SSP (*p* < 0.05). Aquatic sports were inversely associated with SSP(OR = 0.29, 95% CI 0.12–0.71)(p < 0.05). In addition,age-stratified analyses further indicated children (6–11y) exhibited heightened vulnerability to LPA(OR = 5.34, 95% CI 2.62–10.89), both age groups faced elevated risks fromVPA (Children OR = 5.03; Adolescents OR = 3.63), and bilateral sports (e.g., basketball) and aquatic activities universally reduced risk (OR = 0.09–0.19)(p < 0.05).

**Conclusions:**

The amount of physical activity in children and adolescents shows a U-shaped association with SSP, with moderate PA (approximately 54 min/day, 3–4 days/week) being associated with the lowest SSP odds, while low and vigorous PA are associated with higher odds. Participation in water sports and team sports is inversely associated with SSP.These findings highlight potential associations that warrant further longitudinal investigation.

## 1 Introduction

Adolescent idiopathic scoliosis (AIS), a three-dimensional spinal deformity occurring in the ages of 10 and skeletal maturity (1).In recent years, the incidence of scoliosis in children and adolescents continues to rise. The prevalence of scoliosis in Chinese adolescents aged 10–18 years was 1.2%(Cobb angle ≥ 10°) (2).In the latest large-scale spinal screening study, 5.44% of the students were suspected of having scoliosis (3), which is higher than the international screening positive rate of 3.1%(ATR≥5°) (4).AIS profoundly impacts vertebral morphology, intervertebral discs, and peri-spinal structures, contributing to chronic back pain, cardiopulmonary compromise, postural dysfunction, and psychological distress (5–7).

School Scoliosis Screening (SSS) is widely used to identify children and adolescents at risk (8),with an Angle of Trunk Rotation (ATR) of ≥5° commonly defining a positive screening result (SSP) (9).While SSP is not a clinical diagnosis, it serves as an important indicator for early identification and potential referral (10).Due to the different racial and medical models in China, the “Guideline for Adolescent Scoliosis Screening in China (Public Version 2024)” has been issued to guide school scoliosis screening (11).

Physical activity (PA) is a modifiable factor implicated in spinal health (12, 13). Notably, Chinese adolescents exhibit critically insufficient physical activity (PA): only 8.9%–22% meet WHO-recommended thresholds of ≥60 min daily moderate-to-vigorous PA (14–16), falling enhancing spinal musculoskeletal strength, stability, and flexibility (17–20). Inactivity may precipitate paraspinal muscular imbalances and ligamentous stress, driving spinal asymmetry (21)^.^However, most existing studies have used binary (active/inactive) PA classifications, failing to delineate detailed dose-response relationships or optimal thresholds (22).

This study aimed to explore the association between detailed PA parameters (amount, duration, frequency, type) and SSP in a sample of Shanghai schoolchildren. We employed non-linear modeling to characterize dose-response curves and conducted age-stratified analyses. The goal is to provide preliminary evidence to inform future hypothesis-driven research and the development of evidence-based recommendations.

## 2 Manuscript formatting

### 2.1 Study design and participants

**Figure 1:**
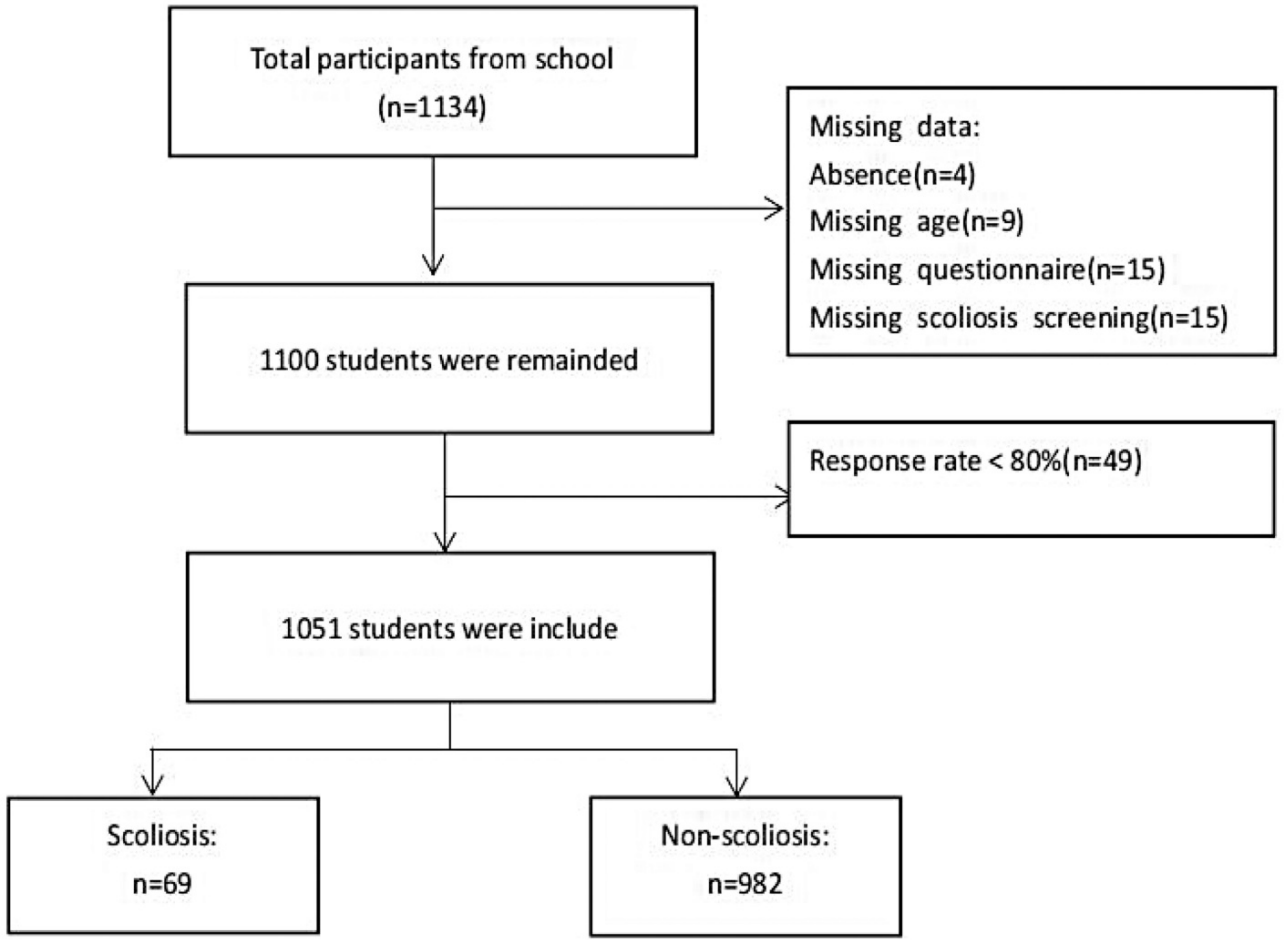
Flow chart of screening subjects.

This was a cross-sectional study. The sample size was calculated for a cross-sectional survey based on an estimated PA compliance rate of 19.7% among adolescents (14). The sample size was calculated for a cross-sectional survey based on an estimated PA compliance rate of 19.7% among adolescents, with a 10% allowable error and a 20% anticipated non-response rate, yielding a minimum required sample of ∼999. A total of 1,051 participants were ultimately enrolled.Participant recruitment employed a three-stage cluster random sampling method. First, four districts (Xuhui, Huangpu, Yangpu, and Fengxian) were randomly selected from all 16 districts of Shanghai. Subsequently, two primary or secondary schools were randomly chosen within each selected district, resulting in a total of eight participating schools. Finally, within each school, students from grades 1 to 11 were sampled by randomly selecting one to two classes per grade, with all students in those classes invited to participate.Inclusion criteria were: aged 6–18 years, enrolled in the selected schools, and providing informed assent/consent. Exclusion criteria were: known diagnosis of scoliosis or other major spinal deformity, physical disability preventing PA participation, or incomplete questionnaire/scoliometer data. After exclusions (*n* = 49 for invalid data), 1,051 participants (511 boys, 540 girls; response rate 95.55%) were included in the analysis. Written informed consent was obtained from all participants and their guardians. The study flowchart is presented in Figure 1.

### 2.2 Scoliosis screening measurements

Screening followed the Guideline for adolescent scoliosis screening in China (public version 2024),and selects children and adolescents aged 6 to 18 for screening according to the guideline (11). A validated electronic scoliometer (JS-101, Jiangsheng, China) was used to perform the Adam's Forward Bend Test. Examiners (*n* = 4) received standardized training on the protocol: participants stood barefoot, feet shoulder-width apart, and slowly bent forward at the waist with arms hanging freely and palms opposed. The examiner placed the scoliometer perpendicular to the spine at each vertebral level from T1 to L5, recording the ATR. The maximum ATR value and its location were noted (23).

An ATR of ≥5° was defined as SSP, a commonly used threshold in school screenings with established sensitivity (24).This approach aligns with public health screening ethics by avoiding unnecessary radiation exposure in a general adolescent population. Participants identified as SSP were notified and advised to seek further clinical evaluation.

### 2.3 Physical activity assessment

Habitual PA was assessed using the validated Chinese version of the Physical Activity Questionnaire for Older Children (PAQ-C) (25, 26). The PAQ-C retrospectively evaluates PA over the past 7 school days via 9 items scored on a 5-point Likert scale, with a final composite score ranging from 1 (low activity) to 5 (high activity). Based on the composite score, participants were categorized into three groups for some analyses: Low PA (LPA: PAQ-C ≤ 2), Moderate PA (MPA: 2 < PAQ-C ≤ 3), and Vigorous PA (VPA: PAQ-C > 3). The PAQ-C has demonstrated good reliability (ICC=0.75–0.82) and validity in pediatric populations (25, 27). Participants also reported the types of sports they participated in, the weekly frequency and average duration per session, and the total years of participation.

### 2.4 Covariate assessment

Based on literature concerning scoliosis risk factors (26, 27), covariates included:

Demographics: Age, sex, body mass index (BMI), educational stage (primary/junior high/high school), parental education level (≤college/>college), family history of scoliosis. Lifestyle/Ergonomics: Handedness, frequency of dairy intake, schoolbag type (backpack/shoulder bag/handbag), self-reported habitual sitting and sleeping postures, frequency of switching seats, and frequency of adjusting desk/chair height. Data on race/ethnicity were not collected as most participants in this setting could not reliably report this information.

### 2.5 Statistical analysis

Data were analyzed using SPSS 27.0 and R 4.1.0. Continuous variables are presented as mea*n* ± standard deviation (SD); categorical variables as frequencies (%). Group differences in baseline characteristics by PA level were assessed using chi-square tests (for categorical variables), with a significance level (*α*) set at 0.05. Multivariable logistic regression was used to estimate adjusted odds ratios (aORs) and 95% confidence intervals (CIs) for the association between PA parameters and SSP. Three models were constructed: Model 1 (unadjusted), Model 2 (adjusted for demographic and family factors: age, sex, BMI, family history, parental education), and Model 3 (further adjusted for lifestyle/ergonomic factors). Statistical significance for regression models was defined as a two-sided *p*-value < 0.05. The dose-response relationships between continuous PA measures (PAQ-C score, daily session duration, weekly frequency) and SSP were modeled using restricted cubic splines (RCS) with 3 knots (located at the 10th, 50th, and 90th percentiles). Missing covariate data (<2% for any variable) were handled using multiple imputation (R mice package, 5 imputations). Subgroup analyses were conducted by stratifying the sample into children (6–11 years) and adolescents (12–18 years) to explore age-specific associations.

## 3 Results

### 3.1 Basic characteristics of participants

A total of 1,051 participants were categorized into low (LPA, *n* = 187), moderate (MPA, *n* = 602), and vigorous (VPA, *n* = 262) physical activity groups based on PAQ-C scores. The prevalence of SSP showed significant associations (*p* < 0.05) with PA level, age group, sport type, years of participation, daily exercise duration, and weekly frequency. The baseline characteristics of participants stratified by PA level (Table 1).

**Table 1:** General demographic characteristics of the subjects.

Characteristics	LPA (n=187)		MPA (n=602)		VPA (n=262)		p-value
	n	%	n	%	n	%	
PAQ-C scores	1.711	0.241	2.424	0.257	3.361	0.271	<0.01
Age	11.13	2.295	10.06	2.334	10.06	2.334	<0.01
Age group							0.05
Ages 6–8	21	9.5%	114	51.8%	85	38.6%	
Ages 9–11	65	17.9%	207	56.9%	92	25.3%	
Ages 12–15	91	22.1%	244	59.2%	77	25.3%	
Ages 16–18	10	18.2%	37	67.3%	8	25.3%	
BMI	18.07	3.104	17.98	3.406	17.23	2.822	0.004
Gender							0.078
female	110	20.4%	298	55.2%	132	24.4%	
male	77	15.1%	304	59.5%	130	25.4%	
Education							0.015
Primary school	119	63.6%	62	33.2%	6	3.2%	
Junior high	139	53.1%	101	38.5%	22	8.4%	
High school	326	54.2%	249	41.4%	27	4.5%	
Family history							0.545
no	153	17.6%	492	56.7%	222	25.6%	
yes	34	18.5%	110	59.8%	40	21.7%	
Education level of father							0.958
Below college	0	0%	7	100%	0	0%	
College or above	3	13.6%	13	59.1%	6	27.3%	
Education level of mather							0.845
Below college	1	33.3%	1	33.3%	1	33.3%	
College or above	6	14.3%	25	59.5%	11	26.2%	
Left and right handedness							0.365
Left hand	6	18.2%	22	66.7%	5	15.2%	
Right hand	181	17.8%	580	57.0%	257	25.2%	
Intake of milk and dairy products							0.345
yes	167	17.6%	541	56.9%	243	25.6%	
no	20	20.0%	61	61.0%	19	19.0%	

### 3.2 Association between physical activity factors and SSP

In the fully adjusted model (Model 3), both LPA and VPA were associated with significantly higher odds of SSP compared to MPA (adjusted OR = 3.30, 95% CI: 1.49–7.35 and OR = 3.65, 95% CI: 1.69–7.90, respectively). Additional factors associated with higher SSP odds included: exercise frequency fewer than three sessions per week (OR = 4.68, 95% CI: 2.13–9.31); cumulative sports participation exceeding two years (OR = 3.37, 95% CI: 1.32–8.60); and session duration shorter than approximately 54 min (OR = 3.43, 95% CI: 1.36–8.60) or longer than 90 min (OR = 2.75, 95% CI: 1.05–7.21). Participation in water sports was associated with lower odds of SSP (OR = 0.29, 95% CI: 0.09–0.98). Detailed associations between various physical activity parameters and SSP are shown in Table 2.

**Table 2:** Association between physical activity factors and SSP.

Variables	No adjustment (Model 1)		Adjusting for family factors (Model 2)		Adjust family, life behavior factors (Model 3)	
	OR	95%CI	OR	95%CI	OR	95%CI
Physical activity level						
MPA	Ref.		Ref.		Ref.	
LPA	2.973*	1.375–6.427*	3.118*	1.428–6.810*	3.304*	1.485–7.351*
VPA	3.035*	1.456–6.327*	3.206*	1.530–6.716*	3.652*	1.688–7.901*
Type of sports activity						
Unilateral exercises	Ref.		Ref.		Ref.	
Gymnastic dance	0.887	0.324–2.429	0.826	0.296–2.306	0.772	0.260–2.293
Water sports	0.110*	0.041–0.291*	0.102*	0.038–0.27*	0.097*	0.036–0.266*
Bilateral exercises	0.176*	0.091–0.340*	0.153*	0.076–0.305*	0.153*	0.075–0.311*
Length of participation						
0–1 years	Ref.		Ref.		Ref.	
2–3 years	0.650*	0.318–1.330*	3.344*	1.359–8.230*	3.373*	1.324–8.597*
≥4 years	0.331*	0.150–0.731*	2.068*	0.936–4.569*	2.227*	0.990–5.009*
Exercise session time						
60–90 min			Ref.		Ref.	
<60 min	3.767*	1.553–9.141*	3.896*	1.591–9.539*	3.428*	1.361–8.597*
>90 min	2.853*	1.134–7.181*	2.756*	1.08–7.035*	2.749*	1.049–7.208*
Exercise frequency						
3–4 times/week	Ref.		Ref.		Ref.	
1–2 times/week	3.261*	1.514–7.026*	2.959*	1.336–6.556*	3.063*	1.37–6.849*
≥5 times/week	1.325	0.611–2.876	1.351	0.61–2.989	1.483	0.652–3.375

* indicates *p* < 0.05.

### 3.3 Dose-response relationships

Restricted cubic spline analyses revealed nonlinear, U-shaped associations. The relationship between the total PAQ-C score and SSP odds reached a nadir at a score of approximately 2.7, with odds increasing at both lower and higher scores (*p* < 0.05). Similarly, the odds of SSP were elevated when the daily exercise session duration was shorter than about 54 min. Exercising less than three times per week was also associated with increased SSP odds.

**Figure 2:**
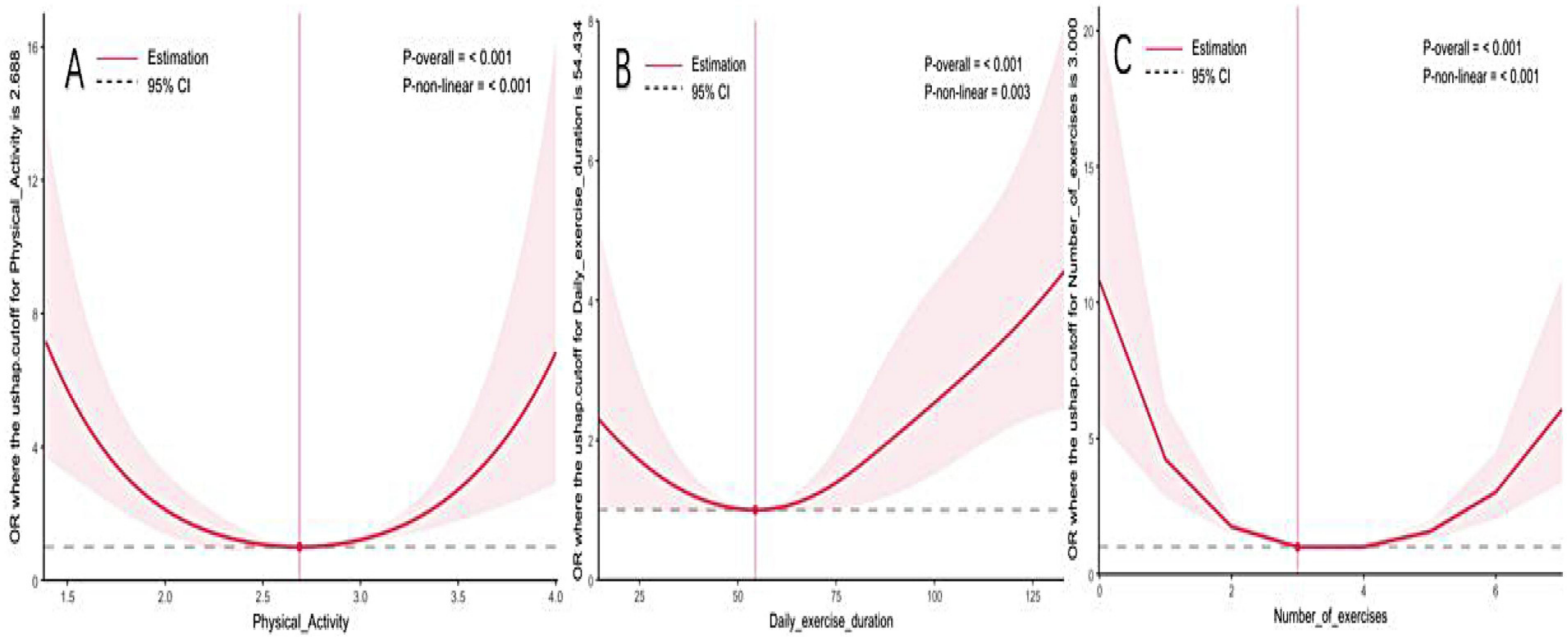
Restricted cubic spline models adjusted for age and sex, showing the dose-response relationship between **(A)** PAQ-C score, **(B)** daily exercise duration, **(C)** weekly exercise frequency and odds of SSP. Shaded areas represent 95% confidence intervals.

 Figure 2 shows the association between SSP and physical activity in children and adolescents.

### 3.4 Age-stratified analyses

**Figure 3:**
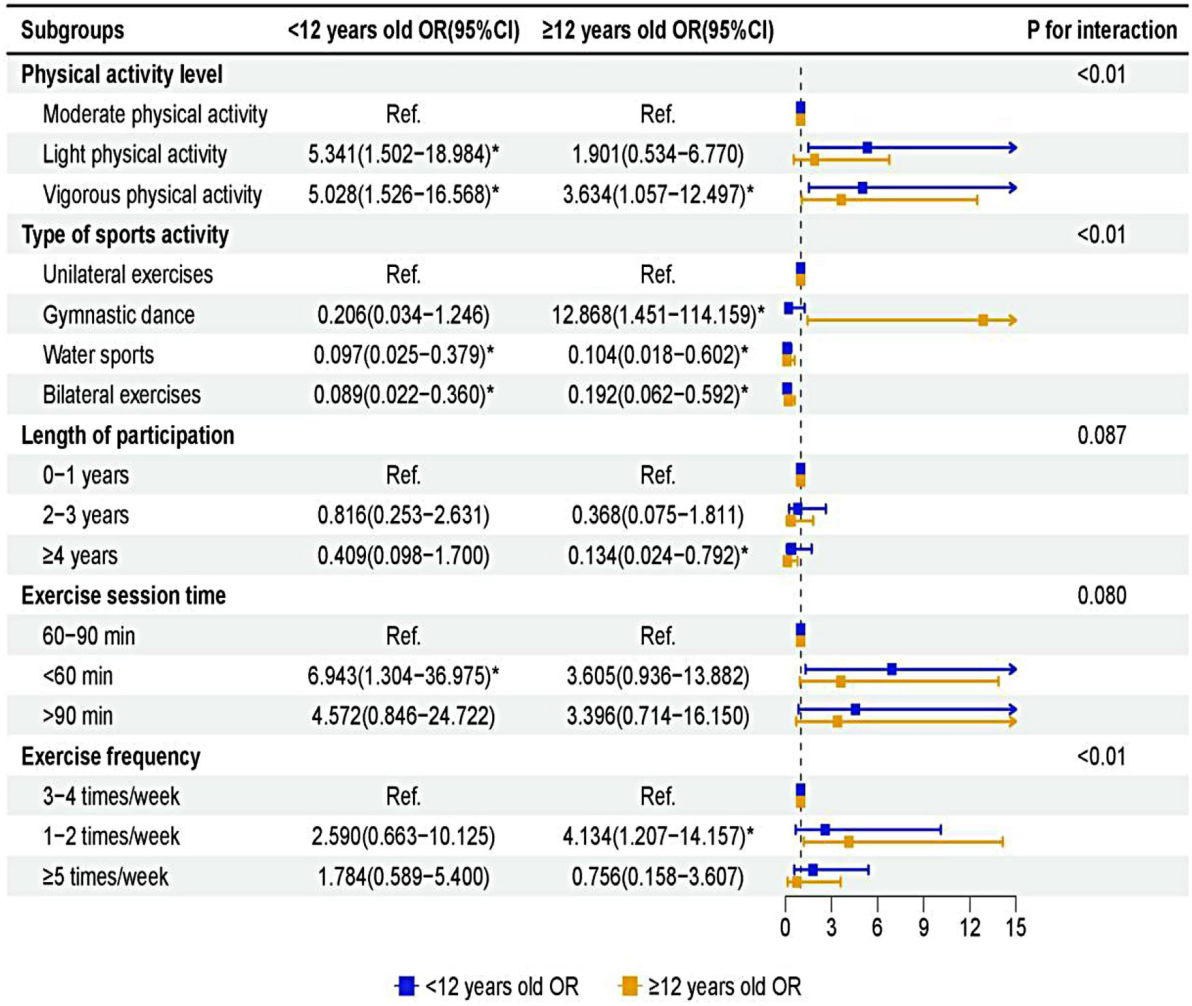
Adjusted relationship between physical activity and SSP, stratified by age group (children: 6–11 years; adolescents: 12–18 years). Odds ratios adjusted for all covariates in Model 3.

Stratification by age group revealed some differential associations. Among children (6–11 years), LPA was associated with markedly higher odds of SSP (OR = 5.34, 95% CI: 1.50–18.98). Both children and adolescents showed elevated SSP odds associated with VPA. Participation in aquatic sports (Children: OR = 0.10; Adolescents: OR = 0.10) and bilateral sports (Children: OR = 0.09; Adolescents: OR = 0.19) was associated with lower SSP odds in both age groups. A session duration of less than 60 min was associated with particularly high SSP odds among children (OR = 6.94, 95% CI: 1.30–36.98). In adolescents, exercising only 1–2 times per week was associated with increased SSP odds (OR = 4.13, 95% CI: 1.21–14.16). Figure 3 shows the age-stratified associations between physical activity factors and SSP.

## 4 Discussion

This cross-sectional study identified a U-shaped association between physical activity levels and SSP among Chinese schoolchildren. The lowest odds of SSP were observed at a moderate PA level, corresponding roughly to 54 min of daily moderate-intensity activity.These findings align with Juskeliene et al.'s observation (28) that children with asymmetric posture had significantly higher rates of low physical activity compared to those with normal posture. The U-shaped relationship may be explained by dual mechanisms: inadequate PA might fail to provide sufficient stimulus for balanced paraspinal muscle development and spinal stability, while prolonged, high-intensity activity might potentially lead to cumulative microstress or fatigue-related postural compromise (7–30). However, due to the cross-sectional design, residual confounding or reverse causation (e.g., individuals with early spinal asymmetries potentially altering their activity patterns) cannot be entirely ruled out, particularly for the vigorous PA group.

The inverse association observed for bilateral sports (e.g., basketball) is consistent with literature suggesting symmetrical neuromuscular engagement may be beneficial. This corroborates Watanabe et al.'s findings (31) where basketball participation was associated with significantly reduced scoliosis risk. Croatian epidemiological data further supports this protective association, showing lower adolescent idiopathic scoliosis rates among football and handball athletes. These activities likely promote symmetric neuromuscular engagement during dynamic movements, counteracting spinal asymmetry development through balanced trunk-pelvic stabilization (32).

The inverse association observed for aquatic activities is consistent with some literature, but the relationship with swimming appears complex and may differ between recreational and competitive contexts.Skoffer et al. (33) observed reduced curve progression with therapeutic swimming programs, while Becker documented (34) elevated scoliosis prevalence in competitive swimmers. This discrepancy may reflect technical proficiency differences, as Zaina et al. (35) proposed that gravitational unloading during aquatic activities might compromise postural control mechanisms. The protective effect in our study population suggests recreational swimming may differ biomechanically from competitive swimming in terms of stroke symmetry and training intensity.

Conversely, unilateral sports were substantially associated with higher odds.Tennis serves generate combined vertical compression and lateral flexion forces, while badminton's arcing technique induces chronic shoulder depression and trunk imbalance (36, 37).Rhythmic gymnastics participation promoted characteristic postural adaptations, correlating with Tanchev et al.'s (38) report of significantly higher scoliosis prevalence in gymnasts compared to peers. These activities impose asymmetric loads on the developing spine, potentially leading to vertebral remodeling over time.

Early sport specialization exceeding four years were associated with higher odds. Song et al. (39)documented significantly higher scoliosis prevalence in table tennis trainees compared to controls, while Tian (40) observed elevated rates in shooters who began training during adolescence. Fencing studies further confirmed progressive curvature increases with training duration, suggesting long-term asymmetric loading induces structural adaptations. These findings highlight the vulnerability of the immature spine to repetitive sport-specific stresses.

Exercise frequency below three weekly sessions significantly increased scoliosis risk, supporting Schwanke et al.'s (41) data showing optimal postural outcomes at this frequency. Session duration exhibited a clear therapeutic window: sessions shorter than 54.43 min or longer than 90 min elevated risk. This aligns with Tobias et al.'s (17) prospective finding that modest increases in daily moderate-to-vigorous activity reduced scoliosis incidence. The inflection point at 54.43 min represents a critical threshold for spinal loading benefits.

The age-stratified results suggest children may be more vulnerable to the effects of very low PA. These findings highlight the potential importance of balanced, moderate physical activity for spinal health in youth and provide a basis for future longitudinal research to investigate causal relationships and refine activity guidelines. In conclusion, this study found that moderate physical activity is associated with the lowest odds of scoliosis screening positivity (SSP) among children and adolescents, whereas both low and vigorous activity levels, as well as certain activity patterns, are associated with higher odds. These associations, observed with SSP as a screening outcome rather than a clinical diagnosis, warrant confirmation in longitudinal studies with clinically confirmed scoliosis.

## 5 Study limitations

This study has several limitations. First, the outcome is SSP (ATR ≥5°), a screening indicator, not a clinically confirmed scoliosis diagnosis. Second, PA was assessed via self-report, subject to recall and social desirability biases. Third, the cross-sectional design precludes causal inference; the observed associations may reflect reverse causality or confounding. Fourth, the sample was from one geographic region in China, which may limit generalizability. Finally, the precision of highly specific threshold values is limited by the measurement methods and should be interpreted as approximate ranges.

## Data Availability

The raw data supporting the conclusions of this article will be made available by the authors, without undue reservation.
